# PGRMC1 Exerts Its Function of Anti-Influenza Virus in the Central Nervous System

**DOI:** 10.1128/Spectrum.00734-21

**Published:** 2021-09-29

**Authors:** Kun Huang, Yufei Zhang, Wenxiao Gong, Yong Yang, Lili Jiang, Lianzhong Zhao, Ying Yang, Yanming Wei, Chengfei Li, Xinglin He, Xiaomei Sun, Zhong Zou, Meilin Jin

**Affiliations:** a State Key Laboratory of Agricultural Microbiology, Huazhong Agricultural University, Wuhan, People’s Republic of China; b College of Veterinary Medicine, Huazhong Agricultural University, Wuhan, People’s Republic of China; c Key Laboratory of Development of Veterinary Diagnostic Products, Ministry of Agriculture, Wuhan, Hubei, People’s Republic of China; Wright State University

**Keywords:** H5N6 virus, CNS, *PGRMC1*, RIG-I, virus replication

## Abstract

The influenza A virus (IAV) infection is usually restricted to the respiratory tract and only rarely enters the central nervous system (CNS) and causes neurological symptoms. However, the roles of host factors involved in IAV infection in the CNS remain largely undetermined. Therefore, we aimed to characterize the host responses to IAV infection in the brain. We isolated a strain of IAV H5N6, which is neurotoxic and highly pathogenic to mice. High-throughput RNA sequencing (RNA-seq) revealed 240 differentially expressed genes in IAV-infected brains. Among the significantly downregulated genes, we focused on the gene encoding progesterone receptor membrane component-1 (*PGRMC1*) and observed that IAV H5N6 infection clearly inhibited *PGRMC1* in both neuroblastoma and glioma cells. Furthermore, treatment with AG205, a *PGRMC1*-specific inhibitor, or *PGRMC1* knockout promoted H5N6 multiplication *in vitro*, while overexpression of *PGRMC1* resulted in opposite effects. Furthermore, AG205 treatment or *PGRMC1* knockout significantly inhibited the retinoic acid-inducible gene I (RIG-I)-mediated interferon beta (IFN-β) signaling pathway and reduced the levels of several antiviral proteins (Mx1 and ISG15). In addition, *PGRMC1*-mediated regulation of IFN signaling relied on inhibition of the expression and ubiquitination of RIG-I. The loss of *PGRMC1* leads to an increased susceptibility of mice (brain and lung) to influenza A virus infection. Conclusively, our results show for the first time that IAV H5N6 downregulates *PGRMC1* expression to contribute to virus proliferation by inhibiting RIG-I-mediated IFN-β production in the brain. These findings may offer new insights regarding the interplay between IAV and host factors that may impact IAV pathogenicity in the brain.

**IMPORTANCE** Central nervous system (CNS) disease is one of the most common extra-respiratory tract complications of influenza A virus (IAV) infections. However, there is still little knowledge about IAV regulating host responses in brain. In this study, we identified progesterone receptor membrane component-1 (PGRMC1) as a novel host factor involved in the replication and propagation of IAV H5N6 in the host brain. We also observed that PGRMC1 antagonism was required for viral evasion from the host immune response during IAV infection via inhibition of the retinoic acid-inducible gene I (RIG-I)-mediated interferon beta (IFN-β) signaling pathway and downstream antiviral gene expression. This study revealed a newly identified regulatory mechanism used by IAV H5N6 to ensure its life cycle in the CNS.

## INTRODUCTION

Influenza A virus (IAV), the causative agent of flu, occasionally causes acute respiratory distress syndrome among humans and many mammalian and avian species. Novel IAVs have emerged via antigenic drift and reassortment events with other IAVs. The highly pathogenic avian influenza virus H5N1 (HPAIV/H5N1) was first detected in a domestic goose in Guangdong Province, China, in 1996 (1). Since then, HPAIV has caused devastating outbreaks repeatedly in wild birds and poultry, as well as sporadic human infections with high mortality. Since 2013, 5 H7N9 influenza epidemic waves have resulted in 1,344 cases, with 511 deaths in China (2). In addition, HPAIV/H5N6 has replaced H5N1 as one of the dominant IAV subtypes circulating in waterfowls and causing human infections in China (3). Therefore, the continuous evolution of the virus represents a long-term threat to public health and the poultry industry.

IAV requires the host cell machinery to support replication of its genome and for the production of new virions (4). Conversely, host cells produce various factors that target different steps in the virus life cycle to restrict virus infection and multiplication. Being part of the first line of defense against infections, type I interferons (IFNs) are key components of the host antiviral innate immune response and are modulators of the adaptive immune response. The innate immune response is triggered in an IAV-infected host to block virus replication and accelerate viral clearance (5). Host recognition of pathogens via pattern recognition receptors (PRRs) is the first step in triggering an immune response. The IAV RNA is recognized by various PRRs, including Toll-like receptors (TLR3, TLR7, and TLR8) retinoic acid-inducible gene I (RIG-I), and NOD-like receptor (NLRP3) (5). These sensors activate signaling cascades resulting in the expression of specific inflammatory cytokines and chemokines (6). Cytokines play various roles, such as direct inhibition of viral replication and activation of the cytolytic functions of T cells, whereas chemokines recruit innate immune cells, such as macrophages, neutrophils, natural killer (NK) cells, and inflammatory monocytes, to the lungs and airways (7). However, viruses have evolved sophisticated mechanisms to evade or counteract host innate immune responses. In particular, genes carried by viruses inhibit host innate antiviral responses by directly targeting IFN gene expression or/and IFN-induced host effector molecules. The nonstructural protein 1 (NS1) is the best characterized and the most important IFN antagonist protein of IAV. NS1 can block type I interferon signaling downstream of RIG-I or may block RIG-I ubiquitination (8). PB1-F2 from IAV strain A/Puerto Rico/8/1934 can interact with mitochondrial antiviral signaling (MAVS) to inhibit interferon production (9).

Central nervous system (CNS) disease is one of the most common extra-respiratory tract complications of IAV infections (10, 11). IAV has been associated with CNS disease since the 1918 H1N1 pandemic, and CNS disease has been observed during all subsequent pandemics, as well as during seasonal epidemics, with occasional detection of IAV in the CNS of humans (12, 13). Zoonotic IAV infects humans only sporadically, but when they do, they are linked frequently to severe and systemic disease (14). HPAIV H5N1 and H7N9 viruses, two recently identified zoonotic IAVs, are associated with CNS disease (14, 15). The HPAI H5N1 virus is possibly the most neurotropic IAV known and has been frequently associated with CNS disease in humans and in other naturally and experimentally infected mammalian species. IAV needs to infiltrate the CNS to infect, replicate, and spread throughout the CNS. IAV reaches the CNS via the olfactory, sympathetic, trigeminal, vagus, and possibly other cranial nerves (16, 17). However, there is still little knowledge about IAV regulation of host responses in the brain.

Progesterone receptor membrane component-1 (PGRMC1) is a 26–28 kDa membrane-spanning protein with a short extracellular domain, a single transmembrane region, and a cytoplasmic domain. The cytoplasmic segment contains a cytochrome P450 b5/heme binding sequence, which can bind to various pharmacological compounds (18). *PGRMC1*, also called Dap1, human progesterone receptor (HPR6.6), and IZA, is a membrane-associated progestin receptor (MAPR) occurring in different species (19). It is highly expressed in the CNS, where it exerts a neurotrophic effect (20). *PGRMC1* has been shown to be involved in multiple cellular functions, such as cholesterol regulation, axonal guidance, endocytosis, and alteration of reproductive behaviors (21–24). However, how *PGRMC1* affects pathogens, especially IAV, remains unclear.

In this study, we aimed to investigate the role of *PGRMC1* in regulating antiviral defense response in brain tissue. Toward this aim, we used a mouse model of IAV infection and human neuroblastoma and glioma cell lines. High-throughput RNA sequencing (RNA-seq) was used to obtain an unbiased profile of the cellular response to IAV H5N6 infection in mice brain. We demonstrated that downregulation of *PGRMC1* is required for IAV replication and propagation in both the human neuroblastoma cell line SK-N-SH and human brain glioma cell line U251. Furthermore, we showed that the *PGRMC1*-specific inhibitor AG205, or *PGRMC1* knockout (KO), functions as an IFN antagonist in IAV infection by negatively regulating type I IFN induction and antiviral gene expression. Our findings reveal a novel mechanism via which IAV H5N6 inhibits host IFN response and emphasizes the vital role of *PGRMC1* in modulating antiviral defenses in the brain.

## RESULTS

### Infection of BALB/c mice with the JX H5N6 virus.

**FIG 1:**
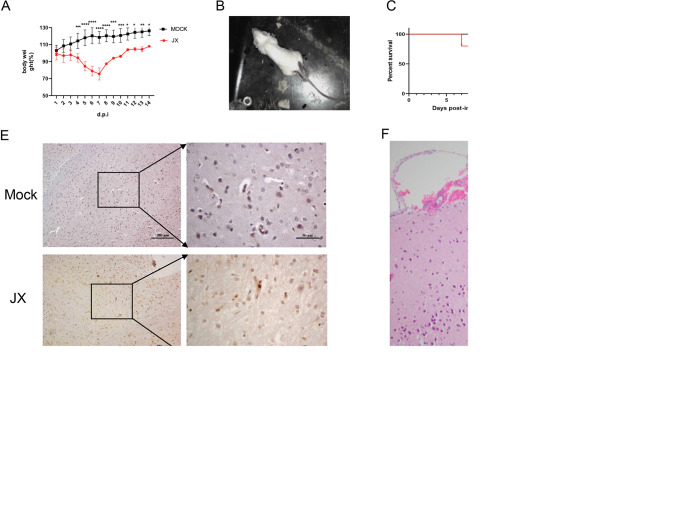
Infection of BALB/c mice (*n* = 11) with H5N6 virus. Mice receiving PBS were used as controls. The mice were monitored for 14 days. (A) Body weight changes were depicted as the percentage of the starting weight of mice (*, *P* < 0.05; **, *P* < 0.01; ***, *P* < 0.001; ****, *P* < 0.0001; using two-way ANOVA). (B) The mice developed neurological symptoms. (C) Survival of infected mice (Mantel-Cox log-rank test). (D) Infectious virion titers in lung and brain were calculated through TCID_50_. (E) Immunohistochemical analysis of the nucleoprotein antigen in brainstem. (F to H) Degeneration and necrosis of neurons and local glial cell nodules in the cerebral cortex. The data shown are representative of three independent experiments with similar results.

Previously, we have demonstrated that HPAIV H5N6 (A/duck/Hubei/WH18/2015, abbreviated as JX) is neurotoxic and highly pathogenic to mice. To establish animal models of brain damage caused by IAV, mice (*n* = 11) were infected intranasally (i.n.) with 50 μl of 10^5^ 50% tissue culture infective dose (TCID_50_), and the mock-treated mice were used as control. Six mice in each group were sacrificed on 3 days postinfection (dpi), and their lung and brains were collected for further study. The remaining five mice in each group were observed daily for the signs of disease and death for 14 dpi. Mice infected with JX showed clearly detectable symptoms of the flu at 3 dpi and showed drastic weight loss (Fig. 1A) [F (1, 98) = 293.2, *P* < 0.0001]. Strikingly, these symptoms had progressed to neurological symptoms, such as balancing problems, hind limb weakness (Fig. 1B), and paralysis by 5 and 6 dpi, and mice began to die by 7 dpi. At the end of the experimental period, only two out of five mice survived (Fig. 1C) (Mantel-Cox log-rank test, *P* = 0.0511). Next, infectious virion titers in brain were determined, and immunohistochemistry (IHC) was performed to investigate whether the brain was successfully infected by the JX virus. Results suggested that the brains of mice were successfully infected. Low viral titers in brain were detected (Fig. 1D). Meanwhile, IHC staining for IAV viral antigen revealed virus-positive neurons in the brains of mice sacrificed on 3 dpi. Virus antigen was detected in multiple areas of the brain, including cerebral cortex, brainstem, and occipital lobe. For representative regions, an image of the cerebral cortex with a single staining of IAV antigen is shown (Fig. 1E). Brain tissue injury caused by the JX virus involves submeningeal hemorrhage, degeneration, and necrosis of neurons and local glial cell nodules (Fig. 1F to H). Collectively, these results demonstrated that the JX virus can infect and spread throughout the CNS, thereby successfully establishing a mouse model for further functional studies.

### Brain transcription profile after JX virus infection.

**FIG 2:**
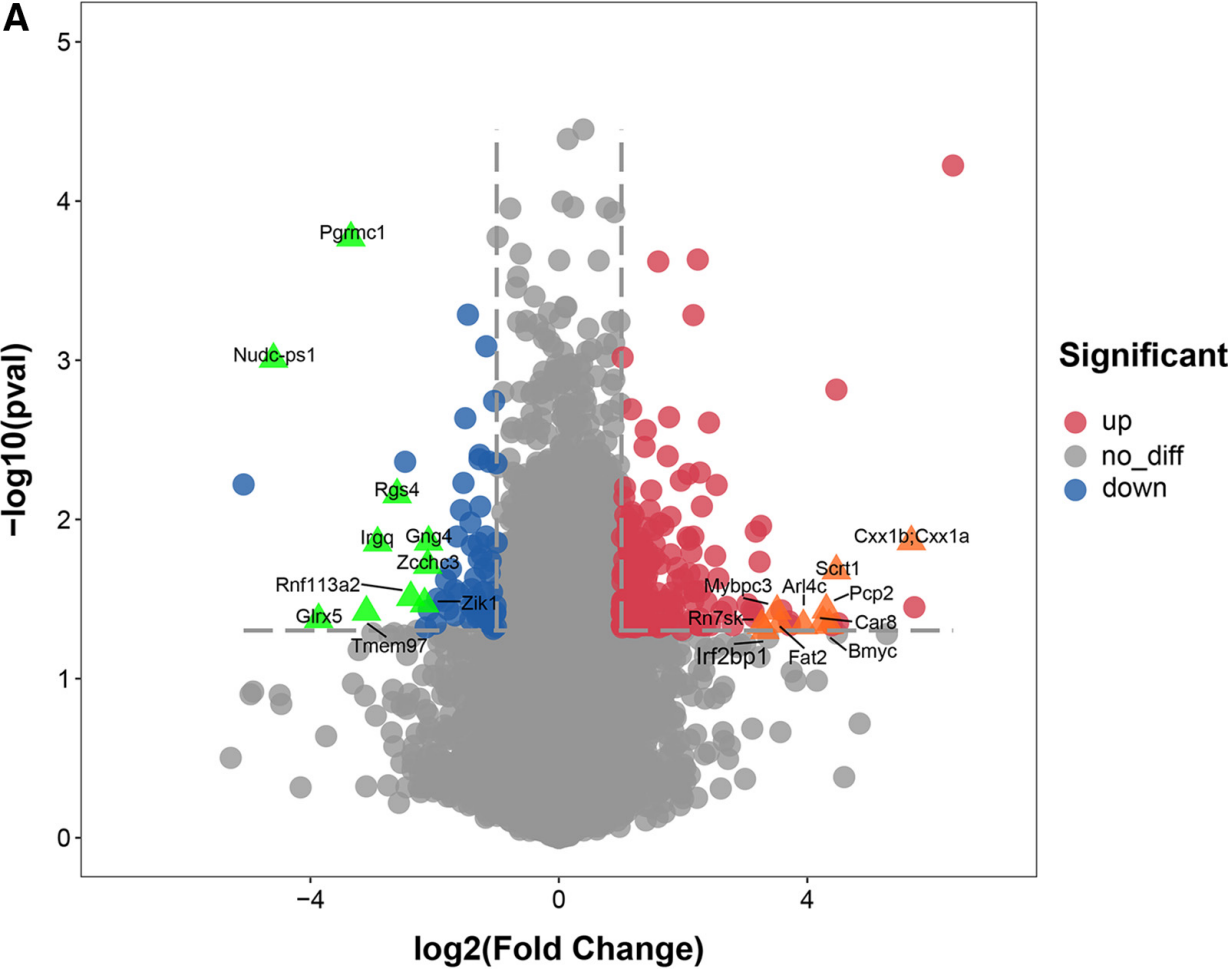
Analysis of the transcriptome profiles of brain tissue after infection with H5N6 virus. (A) Gene volcano plot. (B) Heat map. (C) Scatterplot showing Gene Ontology (GO) and (D) Kyoto Encyclopedia of Genes and Genomes (KEGG) enrichment analysis.

To determine the differential responses of the host brain tissue to infection with JX and mock infection, RNA samples were prepared from whole brains and the complete gene expression profile was compared after whole-transcriptome sequencing (Fig. 2). Histopathological and immunohistochemical examinations showed neuron necrosis, proliferation of glial cells, and viral antigen positivity on 3 dpi. Therefore, brain samples were obtained on this day. A cutoff of >2.0 in fold change and a *P* value of <0.05 were used initially to examine the significantly differentially expressed genes that were up or downregulated following JX infection (Fig. 2). In total, 32,716 genes were detected in the brain, of which 240 were identified as differentially expressed mRNAs between the virus-infected and uninfected mice. Among these genes, 179 were upregulated with log_2_ fold change values ranging from 1.01 to 5.08, while 61 genes were downregulated with log_2_ fold change values ranging from 1 to 4.59. The top 10 upregulated genes listed were *Cxx1b/Cxx1a*, *Scrt1*, *Bmyc*, *Pcp2*, *Car8*, *Arl4c*, *Fat2*, *Mybpc3*, *Rn7sk*, and *Irf2bp1* (Table 1). The top 10 most downregulated genes were *Nudc-ps1*, *Glrx5*, *Pgrmc1*, *Rnf113a2*, *Irgq*, *Rgs4*, *Tmem97*, *Zik1*, *Zcchc3*, and *Gng4* (Table 2). To further assess the connection between gene expression pattern and IAV infection-induced biological processes in the brain, a functional classification of mRNA transcripts and a pathway analysis were performed. These analyses revealed that these genes were involved in regulating neuronal differentiation, ion transmembrane transport, and protein binding. Some of the significant pathways involving the differentially expressed genes were associated with herpes simplex infection, glutamatergic synapse, and chemokine signaling pathway. Among the significantly downregulated genes, we focused on *PGRMC1* to evaluate the role in IAV infection in the brain.

**TABLE 1:** The top 10 most upregulated genes of virus-infected mice compared with control mice

Symbol	Gene name	Log_2_FC	*P* value
Cxx1b;Cxx1a	CAAX box 1A;CAAX box 1B	5.68	0.014
Scrt1	Scratch family zinc finger 1	4.47	0.021
Bmyc	Brain expressed myelocytomatosis oncogene	4.35	0.046
Pcp2	Purkinje cell protein 2 (L7)	4.31	0.037
Car8	Carbonic anhydrase 8	4.25	0.044
Arl4c	ADP-ribosylation factor-like 4C	3.94	0.046
Fat2	FAT atypical cadherin 2	3.56	0.045
Mybpc3	Myosin binding protein C, cardiac	3.51	0.038
Rn7sk	RNA, 7SK, nuclear	3.32	0.05
Irf2bp1	Interferon regulatory factor 2 binding protein 1	3.27	0.046

**TABLE 2:** The top 10 most downregulated genes of virus-infected mice compared with that in the control mice

Symbol	Gene name	Log_2_FC	*P* value
Nudc-ps1	Nuclear distribution gene C homolog (Aspergillus), pseudogene 1	4.59	0.001
Glrx5	Glutaredoxin 5	3.87	0.043
Pgrmc1	Progesterone receptor membrane component 1	3.35	0
Rnf113a2	Ring finger protein 113A2	3.1	0.038
Irgq	Immunity-related gtpase family, Q	2.92	0.014
Rgs4	Regulator of G-protein signaling 4	2.6	0.007
Tmem97	Transmembrane protein 97	2.38	0.031
Zik1	Zinc finger protein interacting with K protein 1	2.16	0.034
Zcchc3	Zinc finger, CCHC domain containing 3	2.11	0.019
Gng4	Guanine nucleotide binding protein (G protein), gamma 4	2.1	0.014

### H5N6 downregulates the expression of *PGRMC1 in vitro*.

**FIG 3:**
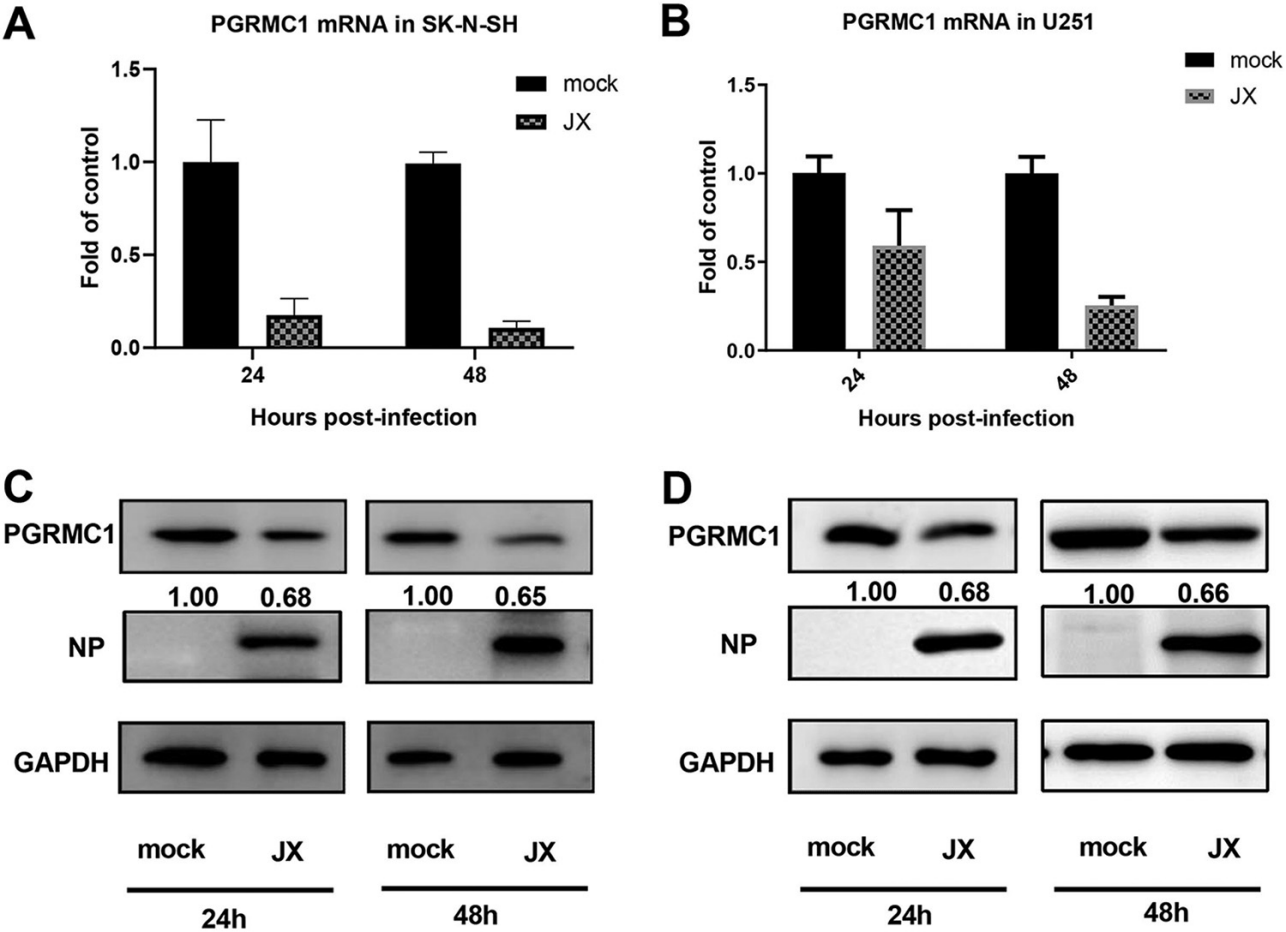
JX inhibited *PGRMC1* expression *in vitro*. *PGRMC1* expression level in SK-N-SH cells (A and C) and U251 cells (B and D). Cells were infected with JX at an MOI of 0.01. Samples were collected at 24 and 48 hpi, followed by qRT-PCR and Western blotting to determine the mRNA (A and B) and protein (C and D) levels of *PGRMC1*. For real-time PCR analysis, the mRNA level was normalized to the GAPDH level. For Western blotting, the band intensities were analyzed using ImageJ, and GAPDH was used as a control in each time point. The relative PGRMC1 levels (PGRMC1/GAPDH) are shown. The data are presented as the mean ± SD from three independent experiments (*, *P* < 0.05; **, *P* < 0.01; ***, *P* < 0.001; ****, *P* < 0.0001; using two-way ANOVA).

It is well known that the nervous tissue is composed of two primary cell types, namely, neurons and glial cells. Neurons transmit nerve messages, while the surrounding glial cells are in direct contact with neurons (24). To verify whether the H5N6 virus can downregulate *PGRMC1 in vitro*, both human neuroblastoma cell line SK-N-SH and human glioma cell line U251 were used. SK-N-SH or U251 cells were infected with JX at multiplicity of infection (MOI; PFU/cell) of 0.01. At 24 and 48 hours postinfection (hpi), *PGRMC1* mRNA and protein levels were assessed using reverse transcription-quantitative PCR (qRT-PCR) and Western blotting, respectively (Fig. 3). Compared with the mock, JX significantly inhibited the production of *PGRMC1* mRNA in SK-N-SH cells after 24 h, which was in agreement with the protein level (Fig. 3A and C) [F (1, 23) = 201.3, *P* < 0.0001]. At 48 hpi, the extent of PGRMC1 protein inhibition by the JX virus was lower than that at 24 hpi. Similar result was observed in U251 cells (Fig. 3B and D) [F (1, 20) = 87.57, *P* < 0.0001]. These results indicated that IAV JX significantly inhibit the expression of *PGRMC1*, which is in agreement with genes identified using RNA-seq.

### PGRMC1 inhibits H5N6 replication *in vitro*.

**FIG 4:**
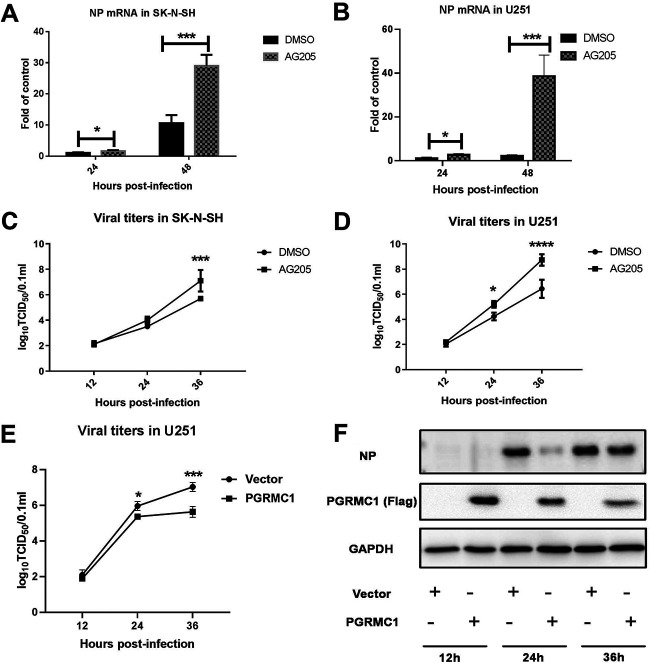
Specific inhibition of PGRMC1 by AG205 increased JX H5N6 virus replication. SK-N-SH cells (A and C) and U251 cells (B and D) were pretreated with 15 μM AG205 or DMSO over 24 h. Cells were infected with JX at an MOI of 0.01. Samples were collected at different time points, and viral NP mRNA and viral titers were determined using qRT-PCR and TCID_50_, respectively. U251 cells (E and F) were transfected with Flag-PGRMC1 and empty vectors. After 24 h, the cells were infected with JX at MOI of 0.01 and expression was detected using Western blotting. TCID_50_ assays (E) for the virus titer of JX were performed to estimate virus multiplication at 12, 24, and 36 hpi. The expressions of PGRMC1 and H5N6 NP protein in U251 cells were detected by Western blot (F). The data are presented as the mean ± SD from three independent experiments (*, *P* < 0.05; **, *P* < 0.01; ***, *P* < 0.001; ****, *P* < 0.0001; using two-way ANOVA).

To better understand the biological role of *PGRMC1* in H5N6 replication, AG205, a putative *PGRMC1*-specific inhibitor (20), was used. SK-N-SH or U251 cells were seeded in 12-well plates and grown to 80% confluence. Then, the cells were treated with 15 μM AG-205 or vehicle (cell culture-grade dimethyl sulfoxide [DMSO]) for 24 h. Cells were infected with JX virus at an MOI of 0.01. Viral nucleoprotein (NP) mRNA levels and viral titers were evaluated at different time points (Fig. 4). NP mRNA levels in the two cell lines significantly increased in the AG205 group compared with that in the control (Fig. 4A) [F (1, 21) = 116.0, *P* < 0.0001] (Fig. 4B) [F (1, 19) = 62.26, *P* < 0.0001]. Next, we observed that AG205 significantly increased the viral titers in U251 24 h and 36 h postinfection (Fig. 4C) [F (1, 12) = 44.47, *P* < 0.0001] (Fig. 4D) [F (1, 12) = 37.78, *P* < 0.0001). However, viral titers significantly increased after AG205 treatment of SK-N-SH cells at 36 h, although no effect on virus replication was evident 24 h after AG205 treatment. Subsequently, we compared the effect of AG205 on viral proliferation in the two cell types and observed that AG205 consistently enhanced the viral titers of IAV JX in the U251 cells compared with that in the SK-N-SH cells. The effect of *PGRMC1* overexpression on viral replication was also determined by transfecting U251 cells with Flag-*PGRMC1*. The efficiency of *PGRMC1* overexpression was determined using Western blotting (Fig. 4F). As observed in the TCID_50_ assays, virus titers at 24 and 36 hpi were lower in the *PGRMC1*-overexpressing groups than in the control group (Fig. 4E) [F (1, 8) = 26.36, *P* = 0.0009]. Taken together, these findings indicated that *PGRMC1* was an IAV JX restriction factor.

### AG205 inhibits the IAV-induced RIG-I-dependent antiviral response.

**FIG 5:**
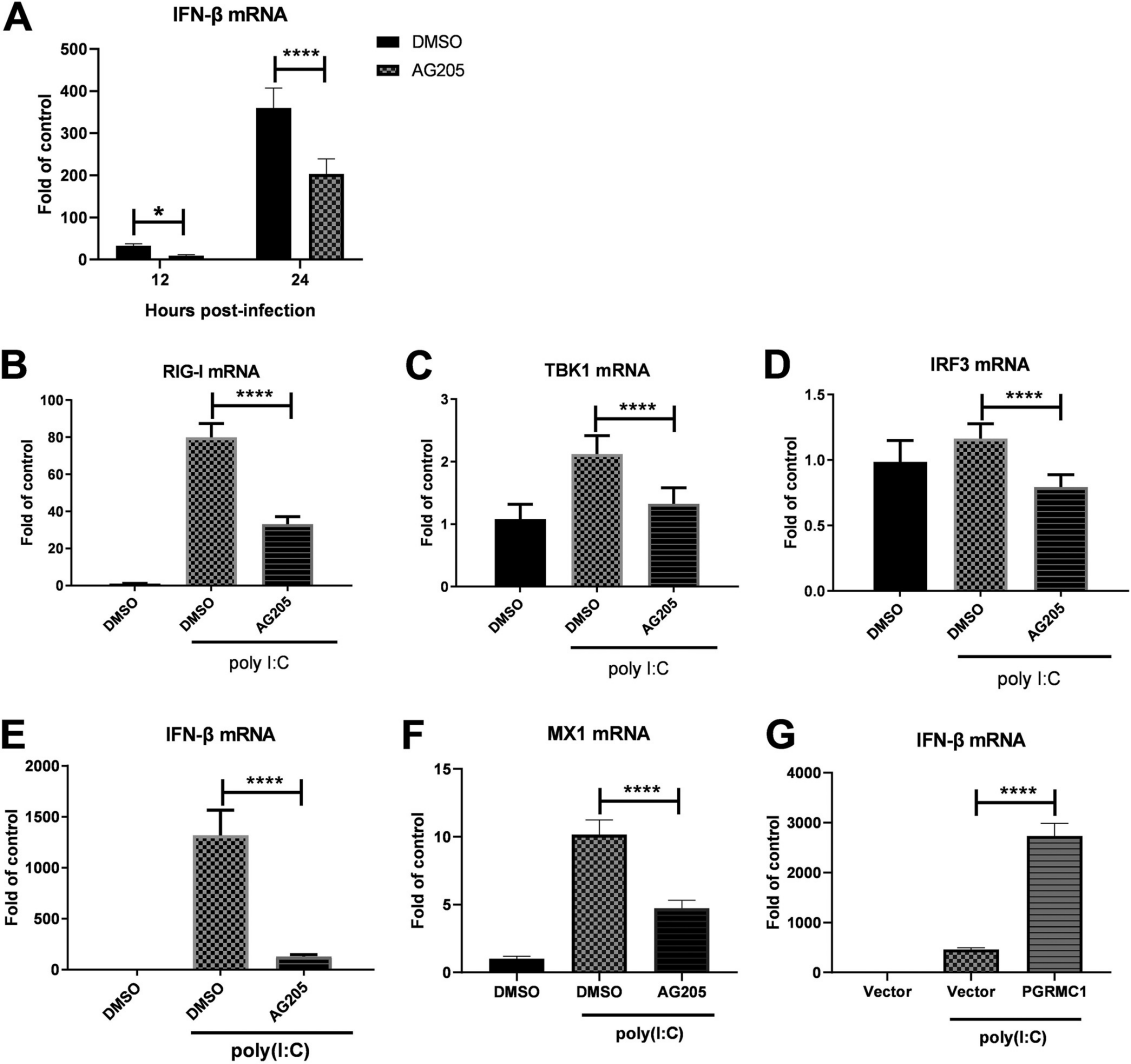
AG205 negatively modulated the IFN-β-inducing pathway. U251 cells were pretreated with 15 μM AG205 or DMSO for 24 h. The cells were infected with JX at an MOI of 0.01 (A) or were stimulated with 100 ng of poly (I·C) (B to F) for 24 h. The mRNA levels of the target genes were detected and normalized to the GAPDH level, and the expression in the DMSO and poly (I·C)-unstimulated groups were set to 1. U251 cells were transfected with Flag-PGRMC1 and empty vectors (G). After 24 h, the cells were stimulated with 100 ng of poly (I·C) for 24 h and IFN-β mRNA levels were detected. The data are presented as the mean ± SD from three independent experiments (*, *P* < 0.05; **, *P* < 0.01; ***, *P* < 0.001; ****, *P* < 0.0001; A was analyzed by repeated measures two-way ANOVA; B to G were analyzed by one-way ANOVA).

**FIG 6:**
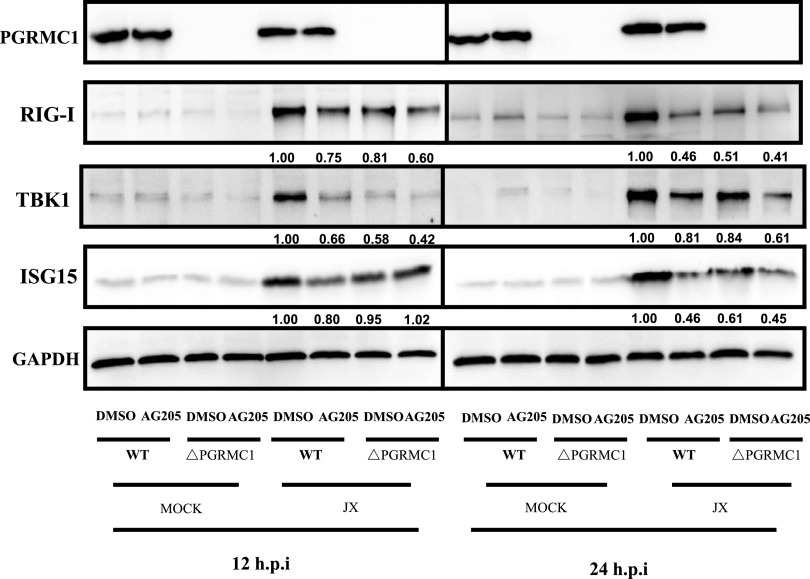
AG205 or *PGRMC1* knockout inhibited the RIG-I-mediated IFN-β signaling pathway. *PGRMC1*-KO U251 cells and wild-type cells were treated with 15 μM AG 205 or DMSO for 24 h. Cells were infected with JX at MOI of 0.01. Samples were collected at 12 and 24 h. IFN-β signaling molecules were detected using Western blotting. The band intensities were analyzed using ImageJ, and GAPDH was used as the control.

AG205 affects virus multiplication more in U251 cells than in SK-N-SH cells. Consistently, the innate immune surveillance is coordinated mainly by glial cells in the CNS (25). These CNS resident cells are assumed to orchestrate the immune response, which assists in combating infections in the brain. Hence, we hypothesized that AG205 may inhibit the IAV H5N6-mediated innate immune response. To test this hypothesis, we evaluated a series of immunological molecules in IAV JX-infected U251 cells, which were incubated with AG205 or DMSO (Fig. 5, Fig. 6). As expected, AG205 significantly downregulated IFN-β induced by IAV JX (Fig. 5A) [F (1, 8) = 126.1, *P* < 0.0001]. In addition, the expression of the downstream effectors of IFN-β, namely, ISG15, was suppressed (Fig. 6). We further observed that AG205 markedly inhibited poly(I·C)-mediated IFN-β induction (Fig. 5B to G). Overall, these observations suggested that AG205 inhibited the type I interferon response.

### AG205 inhibits RIG-I-mediated IFN-β production.

**FIG 7:**
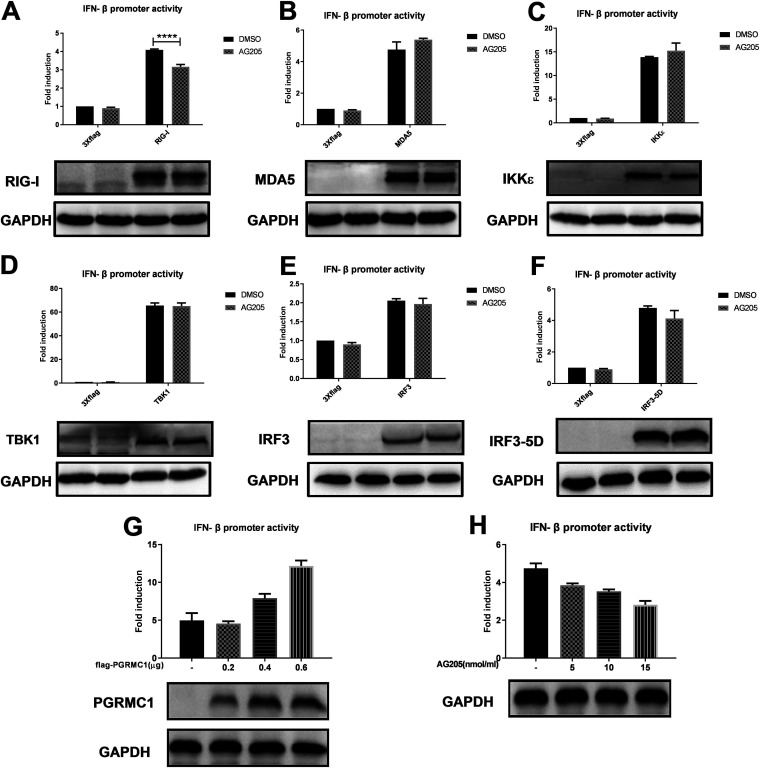
AG205 inhibited IFN-β promoter activity induced by RIG-I-mediated IFN-β signaling molecules. Effect of AG205 or *PGRMC1* on the activation of IFN-β promoter induced by RIG-I (A), MDA5 (B), IKKε (C) TBK1 (D), IRF3 (E), and IRF3-5D (F). HEK293T cells were treated with 15 μM AG 205 or DMSO for 24 h. Then, the cells were transfected with IFN-β-luc, pRL-TK, and the indicated expression plasmids of the signaling molecules. The effect of different doses of *PGRMC1* (G) or AG205 (H) on the activation of IFN-β promoter induced by RIG-I was measured 24 h posttransfection using the luciferase activity assay. The expression level of each signaling molecule was detected using Western blotting with an anti-Flag antibody. The data are presented as the mean ± SD from three independent experiments (*, *P* < 0.05; **, *P* < 0.01; ***, *P* < 0.001; ****, *P* < 0.0001; A to F were analyzed by repeated measures two-way ANOVA; G and H were analyzed by one-way ANOVA).

The RIG-I signaling pathway is essential for IAV recognition. Once activated, RIG-I/MAVS induces the activation of transcription factor IRF3 and NF-κB, ultimately resulting in establishment of the host IFN-mediated antiviral response (5). To investigate how the *PGRMC1* inhibitor AG205 regulates IFN-β, the IFN-β signaling pathway was analyzed using IFN-β luciferase activity assay (Fig. 7). AG205 or DMSO-pretreated 293 cells were cotransfected with the signal molecule expression plasmid, IFN-β promoter luciferase reporter plasmid, and internal control pRL-TK. AG205 markedly inhibited RIG-I-mediated IFN-β production (Fig. 7A) [F (1, 8) = 156.1, *P* < 0.0001]. Interestingly, it did not affect other molecules [MDA5, F (1, 8) = 3.410, *P* = 0.1020; TBK1, F (1, 8) = 0.08015, *P* = 0.7843; IKKξ, F (1, 8) = 1.774, *P* = 0.2196, IRF3, F (1, 7) = 3.168, *P* = 0.1183; IRF3-5D, F (1, 8) = 3.705, *P* = 0.0905] that stimulate IFN-β promoter luciferase activity (Fig. 7B to F). Notably, as shown in Fig. 7H (one-way analysis of variance [ANOVA], F = 46.24, *P* = 0.005), we observed a dose-dependent relationship between AG205 and IFN-β promoter luciferase activity. On the contrary, overexpression of *PGRMC1* increased RIG-I-stimulated IFN-β luciferase activity in a dose-dependent manner (Fig. 7G) (one-way ANOVA, F = 78.66, *P* < 0.0001). Consequently, we concluded that downregulation or functional inhibition of *PGRMC1* reduces the ability of RIG-I to induce IFN-β production in cells infected with IAV H5N6.

### *PGRMC1* knockout promotes IAV replication by inhibiting the RIG-I-mediated IFN-β signaling pathway.

**FIG 8:**
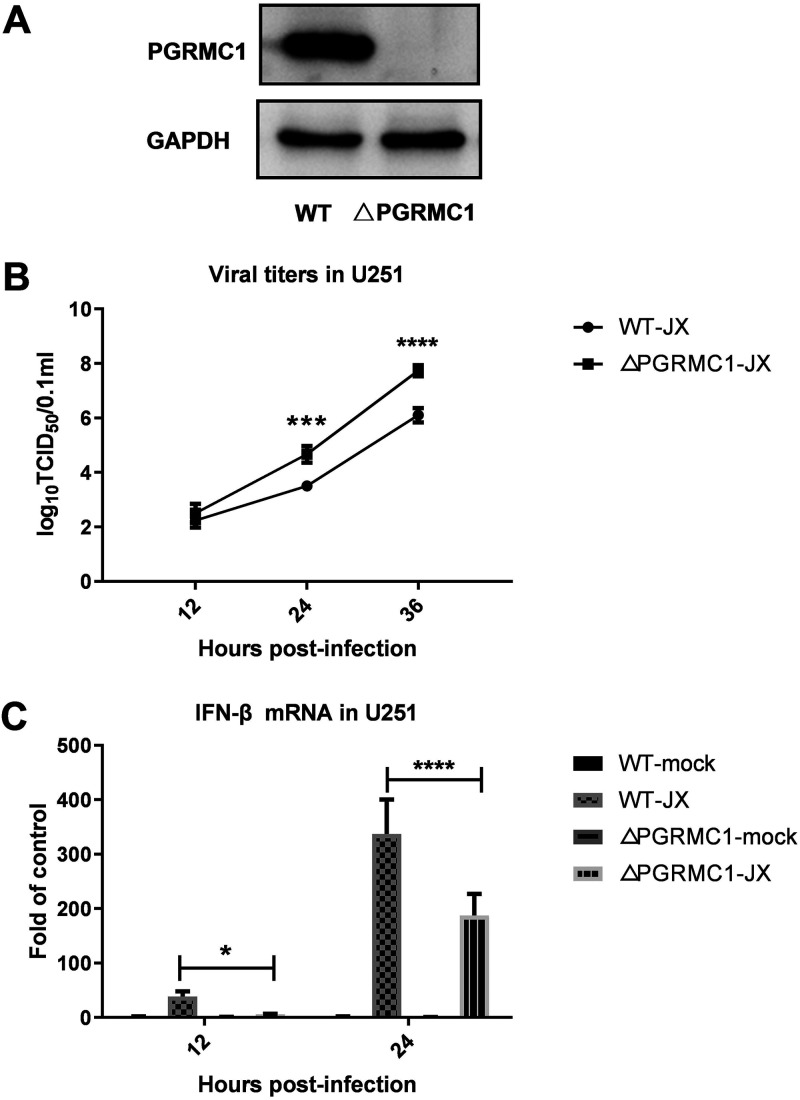
Effect of *PGRMC1* knockout on IAV replication and the RIG-I-mediated IFN-β signaling pathway. Generation of *PGRMC1*-KO U251 cells using the CRISPR-Cas9 system. *PGRMC1* knockout was confirmed using Western blotting (A). *PGRMC1*-KO U251 cells (*ΔPGRMC1*) or wild-type U251 cells (WT) were infected with JX H5N6 virus at MOI of 0.01. Viral titers were determined using TCID_50_ on MDCK cells (B). The mRNA levels of the IFN-β were detected and normalized to the *GAPDH* level (C) (means ± SD from three independent experiments; *, *P* < 0.05; **, *P* < 0.01; ***, *P* < 0.001; ****, *P* < 0.0001; using two way ANOVA).

As the *PGRMC1* inhibitor AG205 has been shown to significantly promote IAV replication, we speculated whether *PGRMC1* knockdown similarly affected the proliferation of IAV H5N6 in U251 cells. Toward this aim, *PGRMC1* was knocked out using the lentiviral clustered regularly interspaced short palindromic repeats (CRISPR)-Cas9 system containing a pair of suitable guide RNAs (gRNAs) driven by two independent U6 promoters on the same plasmid (Fig. 8). After CRISPR-Cas9 gene editing and isolation of individual clones, *PGRMC1* knockout was analyzed using sequencing and Western blot analysis. Three *PGRMC1* KO clones were selected randomly for further research. Our results suggested that CRISPR-Cas9 targeting resulted in the complete loss of the *PGRMC1* protein (Fig. 8A). We evaluated the effect of *PGRMC1* deficiency on IAV JX propagation using a TCID_50_ assay. Indeed, cells with *PGRMC1* knockout produced more infectious viral particles than control U251 cells (Fig. 8B) [F (1, 12) = 70.53, *P* < 0.0001], which is in agreement with the results obtained in cells treated with AG205. Furthermore, the expression of RIG-I (protein level, Fig. 6) and IFN-β (mRNA, Fig. 8C) [F (3, 61) = 185.1, *P* < 0.0001] in JX-stimulated cells decreased. Alternatively, the IFN-β upstream factors (RIG-I, and TBK1) and IFN-β downstream effector (ISG15) were also significantly downregulated (Fig. 6). These results further confirmed that *PGRMC1* knockout promoted virus replication by efficiently suppressing RIG-I-mediated IFN-β production.

### *PGRMC1* knockout antagonizes RIG-I ubiquitination.

**FIG 9:**
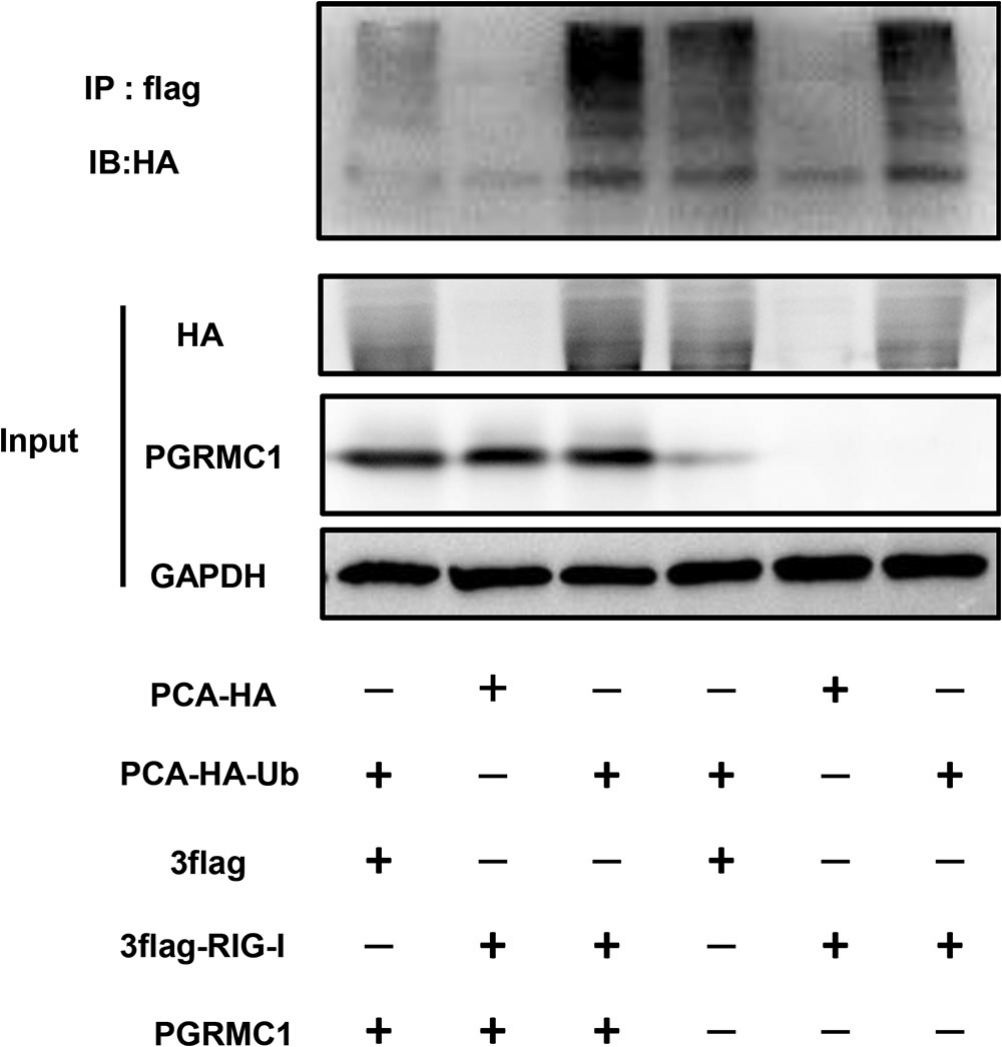
*PGRMC1* knockout inhibited RIG-I ubiquitination. *PGRMC1*-KO U251 cells and wild-type cells were transfected with Flag-RIG-I and pUb-HA. After 24 h, the cells were infected with JX at an MOI of 0.01. After 24 h, the cells were subjected to anti-Flag immunoprecipitation and immunoblotting with anti-HA to monitor RIG-I ubiquitination.

RIG-I exists in an inactive closed conformation under normal conditions. RIG activation requires ubiquitination induced by the tripartite motif 25 (TRIM25) ubiquitin E3 ligase. Hence, we next addressed whether *PGRMC1* knockout specifically suppresses RIG-I ubiquitination (26). Toward this aim, we assessed RIG-I ubiquitination in *PGRMC1*-KO U251 cells or wild-type (WT) U251 cells transfected with the pCAGGS-HA-Ub vector or the empty vector along with or without p3×Flag-RIG-I, followed by infection with JX. Samples were subjected to anti-Flag immunoprecipitation and immunoblotting with an anti-hemagglutination (HA) tag antibody to monitor RIG-I ubiquitination. As shown in Fig. 9, *PGRMC1* knockout markedly inhibited RIG-I ubiquitination, as shown by comparison of lanes 3 and lane 6. These results revealed collectively that the *PGRMC1* knockout inhibited RIG-I ubiquitination in U251 cells, thereby suppressing RIG-I signal transduction.

### *PGRMC1* knockout promotes IAV replication in brain and lung of mice.

**FIG 10:**
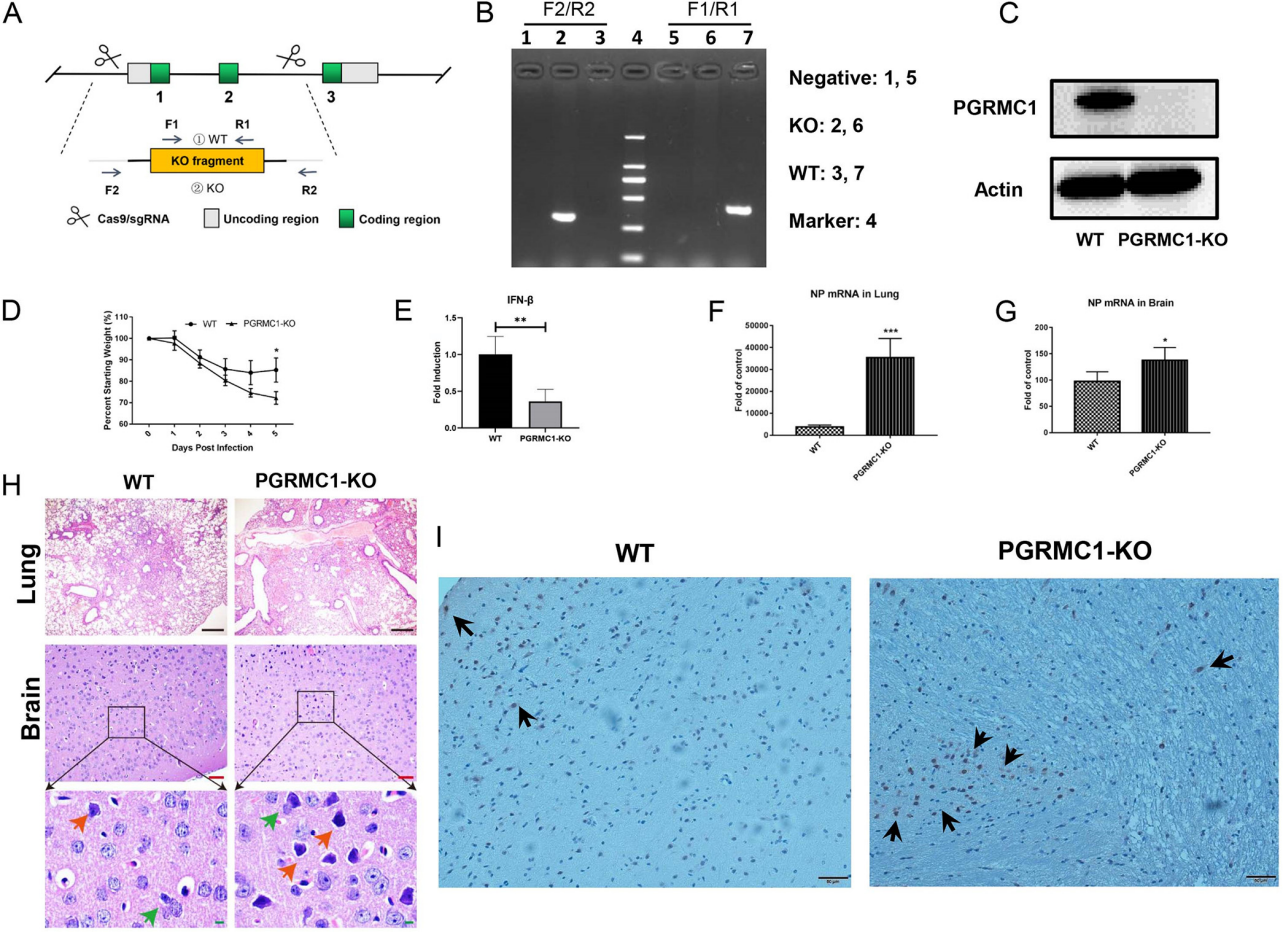
*PGRMC1* knockout promoted IAV replication in mice. The *PGRMC1* KO mice were made by using the CRISPR-Cas9 technique (A). The analyses of protein expressions in WT and *PGRMC1* KO mice by PCR (B) and Western blotting using the antibody against PGRMC1 and actin (C). C57BL/6J WT and *PGRMC1* KO female mice (*n* = 5) aged 4 to 6 weeks were infected intranasally with 50 μl of 10^5^ TCID_50_ H5N6 virus. Body weights were monitored daily for 5 days (D), and three mice in each group were euthanized at 5 dpi. IFN-β mRNA in lung (E) and viral NP mRNA in lung (F) and brain (G) were determined through qRT-PCR. Hematoxylin and eosin (H&E) staining of lung and brain sections from WT and *PGRMC1* KO mice infected H5N6 virus (H). Astrocytic hyperplasia, red arrow; neuronophagia, green arrow. (I) Viral NP proteins (black arrows) were detected in the cerebral cortex of WT and *PGRMC1* KO mice. Scale bars (black), 200 μm. Scale bars (red), 50 μm. Scale bars (green), 10 μm. (*, *P* < 0.05; **, *P* < 0.01; ***, *P* < 0.001).

To further confirm the role of *PGRMC1* in IAV infection in the brain, we utilized the *PGRMC1* KO and wild-type (C57BL/6J) mice (Fig. 10). *PGRMC1* KO mice were generated using the CRISPR-Cas9 technique (Fig. 10A) and were verified by PCR (Fig. 10B) and Western blotting (Fig. 10C). The *PGRMC1* KO and WT mice were infected with JX IAV strain, and 5 days later, mice were sacrificed to collect the brain and the lung. Both KO and WT mice started losing weight from day 2 after virus inoculation (Fig. 10D). By day 5, WT mice had lost 15% of their body weight. In contrast, the KO mice lost a significantly higher amount of their body weight, namely, 28%, by day 5. The qRT-PCR analysis revealed that IFN-β mRNA level in KO mouse lung was significantly lower than that in wild-type mouse lung (Fig. 10E). More interestingly, the NP mRNA level in KO mouse lung and brain was significantly higher than that in WT mouse lungs (Fig. 10F and G). Histopathologic analysis showed that the lungs of *PGRMC1* KO mice displayed more severe pathologies on day 5 postinfection than that of WT mice (Fig. 10H), including inflammatory cell infiltration, and deciduous cells in the bronchial lumen, even the alveoli, almost disappeared. In contrast to the brain in WT mice, more astrocytic hyperplasia, neuronophagia phenomenon, and neuronal cell necrosis were observed in the brain tissues from *PGRMC1* KO mice. The immunohistochemical assays show a similar result in NP mRNA levels in the brain (Fig. 10I). These results further confirmed that *PGRMC1* knockout promotes virus replication and leads to more serious damage *in vivo*.

## DISCUSSION

IAV is known to target the respiratory system, while some particular subtypes of IAV can infect the brain and cause serious damage to the CNS. Multiple routes of IAV invasion into the CNS after intranasal exposure have been reported. In this study, we isolated a strain of HPAIV H5N6 virus from a duck, which showed high fatality and pathogenicity to mice. Some infected mice developed typical neurological symptoms, such as balancing problems, hind limb weakness, and paralysis. Surprisingly, although the mice survived until the completion of the experiment, hind limb paralysis did not improve. This observation was in accordance with the results of Hosseini et al., who reported that neuroinflammation caused by nonneurotropic and neurotropic IAV induces long-lasting impairments in hippocampal neuronal morphology and synaptic properties and cognitive function in adult animals (27). The viral loads in the brain of mice in the infected group were examined using a TCID_50_ assay. These results confirmed that the JX virus can infect, replicate, and spread throughout the CNS. This finding agrees with the observation that viruses of the HPAIV H5 subtype are associated more frequently with CNS disease in humans (28).

The molecular mechanisms underlying the involvement of host factors in IAV infection in CNS remain obscure. Here, we identified 240 genes in the IAV-infected mouse brains using a RNA-seq screen. These genes were involved in herpes simplex infection, glutamatergic synapse, and chemokine signaling pathway. Among these identified genes, *H2-T23*, *H2-T24*, *Ifi1*, *Irf7*, and *Stat2* were involved in other virus infections, such as those caused by chikungunya virus, Duvenhage virus, and Zika virus. Zhao and coworkers observed that five virus-related proteins (TGTP, IFIT3, IFIT3-L, LCP1-1, and LCP1-2) were upregulated within 1 dpi in the lethal virus-inoculated group but did not show appreciable upregulation until 3 dpi in the nonlethal group. Their results suggest that proteomic approaches can distinguish the lethality of the influenza strain in a mouse model within 24 h of infection (29). Interestingly, the interferon-responsive gene *Ifit3*, which is reported to be involved in cognitive decline in aged mice (30), was specifically upregulated in the hippocampus of H7N7-infected mice and is expressed mainly in granule cells (31).

*PGRMC1* participates in cellular processes relevant to CNS diseases, including neuroprotection, axonal migration, and mitochondrial protection. Furthermore, it is expressed highly in the brain. However, the relationship between *PGRMC1* and virus infection in the brain remains uninvestigated. Here, we observed that HPAIV H5N6 significantly inhibited *PGRMC1* expression in the brain. This phenomenon has further been demonstrated in SK-N-SH and U251 cells. Next, we investigated the effect of *PGRMC1* on H5N6 and observed that AG205-mediated inhibition of *PGRMC1* increased viral titers in these two cell lines. Although AG205 was advantageous for the proliferation of H5N6 in SK-N-SH and U251 cells, it promoted viral replication more in U251 cells than in SK-N-SH cells. U251 cells are derived from glial cells, which play a key role in regulating the immune responses in CNS. In addition, one of the first lines of host immunological defense against viruses involves the IFNs. Therefore, we hypothesized that AG205 treatment or *PGRMC1* knockout may inhibit the H5N6-mediated IFN signaling pathway. As expected, AG205 treatment or *PGRMC1* knockout inhibited the type I interferon response dramatically, implying that *PGRMC1* is a host factor restricting IAV JX infection in the CNS.

IAV genomes consist of eight negative-sense single-stranded RNA segments (vRNA) that encode at least 14 viral proteins (32). Hence, we aimed to identify the gene(s) involved in *PGRMC1* inhibition. To this end, eight viral gene segments were cloned individually in the pHW2000 expression vector. However, unfortunately, we observed that no single IAV JX gene segment was able to significantly downregulate *PGRMC1* (Fig. S1). This finding indicated that a single gene or protein is not sufficient for inhibiting *PGRMC1* and that downregulation of *PGRMC1* is relevant for the biological characteristics of H5N6 virus.

During coevolution with hosts, IAV has developed multiple strategies to escape the host antiviral innate immunity, especially via blockade of the IFN system. Several IAV proteins have been well characterized as IFN antagonists, which antagonize the host IFN response at different steps. IAV antagonizes IFN response via various strategies, which involve viral proteins and host factors. For example, NS1 of IAV inhibits the activation of IRF3 and IFN-β transcription. In addition, PB2 and PB1-F2 interact with MAVS, which represses IFN-β expression (9). In this study, H5N6 downregulated *PGRMC1* in the brain, which strongly suppressed the activation of the RIG-I-mediated IFN-β signaling pathway. Furthermore, AG205 treatment or *PGRMC1* knockout inhibited not only RIG-I expression stimulated by the H5N6 virus but also RIG-I ubiquitination. These results suggest that IAV-induced downregulation of *PGRMC1* is involved in controlling RIG-I signaling, which is beneficial for the virus infection in the CNS. However, the mechanisms by which downregulated *PGRMC1* inhibits the expression and ubiquitination of RIG-I remain unclear. Possibly, downregulated *PGRMC1* perturbs the interaction between RIG-I and its E3 ligase, TRIM25, which warrants further investigations. Previous studies have assumed that *PGRMC1* can alter its subcellular location under certain circumstances. Peluso suggested that as a transcription factor, *PGRMC1* is involved in the regulation of gene transcription (33).

Microglia and astrocytes are the CNS-resident cells involved most prominently in early innate responses to injury, autoimmune attack, or infection (25). Unfortunately, pathogens can induce devastating inflammatory damage to the CNS, leading to rapid mortality or long-term neurological disabilities. Nevertheless, some viruses may survive as latent infections in conducive environments, provided they become dormant and do not proliferate. Thus, the CNS “protects” these pathogens.

In summary, we identified *PGRMC1* as a novel host factor involved in the replication and propagation of IAV H5N6 in the host brain. We also observed that *PGRMC1* was required for evasion from the host immune response during IAV infection via inhibition of the expression and ubiquitination of RIG-I, thereby blocking activation of the RIG-I-mediated IFN-β signaling pathway and downstream antiviral gene expression. Overall, our study revealed a newly identified regulatory mechanism used by IAV H5N6 to ensure its life cycle in the CNS. However, further studies are necessary to explore more precisely the specific mechanism of the H5N6 virus that downregulates *PGRMC1* expression.

## MATERIALS AND METHODS

### Ethics statement.

All animal experiments were approved by the Research Ethics Committee, Huazhong Agricultural University, Hubei, China (HZAUMO-2016-022), and were performed in accordance with the Guidelines for the Care and Use of Laboratory Animals of the Research Ethics Committee, Huazhong Agricultural University, Hubei, China.

### Cells and viruses.

The human neuroblastoma cell line SK-N-SH, human brain glioma cell line U251, and the Madin-Darby canine kidney cell line (MDCK) were cultured in Dulbecco’s modified Eagle’s medium (HyClone, Logan, UT, USA) supplemented with 10% fetal bovine serum (PAN Biotech) and 100 U/ml penicillin-streptomycin (Thermo Fisher Scientific, Inc.). HEK293T cells were cultured in RPMI 1640 medium (Invitrogen). The IAV strain used in this study was the A/duck/Hubei/WH18/2015(H5N6) (JX) (GenBank accession number KX652135) strain isolated in our laboratory from an infected duck lung. H5N6 was grown in 9-day-old embryonated eggs. The titers of IAV in culture media were determined using the 50% tissue culture infective dose (TCID_50_) and plaque assays in MDCK cells.

### Mouse infection.

Female BALB/c mice aged 4 to 6 weeks were purchased from the Center for Animal Disease Control, Hubei Province, China. Mice (*n* = 11) were infected intranasally (i.n.) with 50 μl of 10^5^ TCID_50_, while naive control mice were inoculated with phosphate-buffered saline (PBS). Six mice in each group were sacrificed on 3 dpi, and their brains were collected for further study. For pathogenicity analysis, three brains was split in half. The right hemisphere was collected in 1 ml of PBS supplemented with antibiotics (100 U/ml penicillin-streptomycin), and the left hemisphere was placed in formalin. For transcriptomic analysis, three other brains were harvested and stored in TRIzol reagent (Invitrogen, Carlsbad, CA, USA). The remaining five mice in each group were observed daily for the signs of disease and death for 14 days postinfection (dpi).

### Immunohistochemistry (IHC).

The fixed brains were paraffin embedded, sectioned, and stained with a rabbit anti-NP-specific monoclonal antibody (GeneTex). Goat anti-rabbit immunoglobulin conjugated to peroxidase (Maxim Bio, Fujian, China) was used as the secondary antibody. The sections were screened using an Olympus BX51 microscope coupled to a camera.

### Plasmid construction.

The IFN-β-luc, RL-TK, RIG-I, MDA-5, TBK-1, IκB kinase ε (IKKε), IFN regulatory factor (IRF3), IRF3-5D, and pUb-HA plasmids were provided kindly by Zhengfan Jiang (Peking University) (4). *PGRMC1* was cloned in the p3×Flag-CMV-14 vector. A lentiviral vector encoding EGFP-Cas9, the packaging plasmid pMD2.G, and the envelope plasmid psPAX2 were provided kindly by Lisheng Zhang (Huazhong Agricultural University). The *PGRMC1* guide RNA was cloned in the lentiviral vector. Plasmids expressing Flag-RIG-I were amplified from HA-RIG-I and cloned into p3FLAG-CMV-14 vectors.

### RNA sequencing and gene expression analysis.

Total RNA was isolated and purified using the TRIzol reagent (Invitrogen, Carlsbad, CA, USA) following the manufacturer’s procedure and was dispatched to the LC-Bio Corporation (Hangzhou, Zhejiang, China) for microarray analysis. The reads were assembled using StringTie. Next, transcriptomes were merged to reconstruct a comprehensive transcriptome using perl scripts. In this study, StringTie was used to determine the expression level of mRNAs by calculating fragments per kilobase per million (FPKM). The differentially expressed genes were selected with log_2_ (fold change) of >1 or log_2_ (fold change) of <−1 and with statistical significance (*P* value of <0.05) by using R package Ballgown.

### RNA extraction and reverse transcription-quantitative PCR (qRT-PCR).

Total RNA was extracted from brains or cells using TRIzol according to the manufacturer’s instructions. Next, the mixed genomic DNA was digested with DNase I and 1-μg RNA was reverse transcribed using the reverse transcriptase (TaKaRa Biotechnology, Dalian, China) from avian myeloblastosis virus with oligonucleotide (dT) 18. cDNA (0.5 μl) was used as the template for qRT-PCR. In addition, the reaction mixtures contained 5-μl 2× SYBR green master mix (Roche, Indianapolis, IN, USA), 0.25 μl of each primer (10 mM), and 4-μl ultrapure water. The assay was run on an ABIViiA 7 PCR system (Applied Biosystems, Waltham, MA, USA). The expression of each gene was normalized to that of glyceraldehyde 3-phosphate dehydrogenase (GAPDH) as a control.

### Luciferase reporter assays.

HEK293T cells cultured in 24-well plates were incubated with the *PGRMC1* inhibitor AG205 (Sigma-Aldrich) or dimethyl sulfoxide (DMSO) for 24 h. The cells were transfected with IFN-β-luc (200 ng/well) and the internal control pRL-TK (10 ng/well). After 24 h, the cells were transfected with poly(I·C) (200 ng/ml) and incubated for 12 h, followed by analysis of cell lysates for luciferase activity with a dual luciferase reporter assay kit (Promega) according to the manufacturer’s instructions. HEK293T cells seeded in 24-well plates were cotransfected with the expression plasmid harboring *PGRMC1* or the empty vector IFN-β-luc (200 ng/well) and the internal control pRL-TK (10 ng/well), together with the adaptor molecules (RIG-I, MDA-5, TBK-1, IKKε, IRF3, and IRF3-5D) of each signaling pathway. Luciferase activity was measured as mentioned above at 24 h posttransfection.

### Western blotting.

The cells were washed with cold PBS and lysed on ice in Tris lysis buffer (Cell Signaling Technology, Danvers, MA, USA) containing 1% EDTA-free protease inhibitors (Roche). To remove cell debris, the cell lysates were centrifuged at 12,000 rpm for 15 min at 4°C. The supernatant was resuspended in 1× sodium dodecyl sulfate (SDS) loading buffer, was resolved using SDS-polyacrylamide gel electrophoresis (SDS-PAGE), and transferred to nitrocellulose membranes (GE). The membrane was first probed using certain primary antibodies and then with horseradish peroxidase-conjugated goat anti-mouse or anti-rabbit secondary antibodies (Abcam; Cambridge, MA). Finally, a chemiluminescence imaging system (DNR, Neve Yamin, Israel) was used for the analysis, and images were acquired.

### Generation of *PGRMC1* knockout U251 cells.

*PGRMC1*-KO U251 cells were generated using the clustered regularly interspaced short palindromic repeats (CRISPR)-Cas9 system and lentiviral vectors as described previously (34). The single guide RNA sequence targeting the human *PGRMC1* (5′-GCTCTACAAGATCGTGCGCG-3′) was cloned in the BsmBI site of the EGFP-Cas9 lentiviral vector. The EGFP-Cas9 lentiviral vector psPAX2 and pMD2.G were cotransfected in 293T cells. The cell supernatant was harvest 48 h posttransfection. Then, U251 cells were incubated with the viral supernatant or the empty vector lentivirus. Finally, the monoclonal cells were sorted using flow cytometry and seeded in a 96-well plate to generate clonal cell lines. *PGRMC1* knockout was confirmed via sequencing and Western blotting.

### Coimmunoprecipitation (co-IP) assay.

Wild-type (WT) or *PGRMC1*-KO U251 cells were transfected with pCAGGS-HA-Ub together with p3×Flag-RIG-I. At 48 h posttransfection, the cells were lysed with buffer for Western blotting and IP (Beyotime, Beijing, China) at 4°C for 30 min. The supernatants were collected and incubated overnight with anti-HA magnetic beads (Bimake, Beijing, China) at 4°C. Then, the beads were washed thrice with lysis buffer and eluted with 1× SDS loading buffer. Samples were tested using Western blotting.

### H5N6 virus replication in WT and *PGRMC1* KO mice.

The *PGRMC1* KO mice were made by GemPharmatech Inc. by using the CRISPR-Cas9 technique. The gRNA sequences to deplete *PGRMC1* are as follows: GGCGCTTAGGTGGCCGCGAA (gRNA1) and GGGCCCCCTATGTAGCCTCG (gRNA2). The 4,525-bp DNA fragment was depleted by these two gRNAs, resulting in a frameshift for the rest of the cDNA. For routine PCR genotyping of *PGRMC1* KO mice, the following primer pair were designed to amplify a 414-bp (F1/R1) PCR product from wild-type mice and a 394-bp (F2/R2) product from mutant alleles: F1, CCTCGTCATCTGGTTGTATTTGC; R1, GCTAGCATCTACGACTGTGCTGTAC; F2, TGTCTGCGAGCTGCACTATGATC; and R2, TGCAGACACCATTGTTTGCTG.

C57BL/6J WT and *PGRMC1* KO female mice (*n* = 5) aged 4 to 6 weeks were infected intranasally with 50 μl of 10^5^ TCID_50_. Body weights were monitored daily for 5 days, and three mice in each group were euthanized at 5 dpi. Lungs and brains were collected for further studies. For lungs or brains, the right half was collected in 1-ml PBS supplemented with antibiotics (100 U/ml penicillin-streptomycin) and the left was placed in formalin for histopathology. Viral RNA copies in lungs and brains were determined with qRT-PCR.

### Laboratory facility.

All experiments involving live viruses were performed in a biosafety level 3 (BSL3) facility in accordance with the institutional biosafety manual. The animals were housed in negative-pressure isolators with high-efficiency particulate air filters in the BSL3 facility.

### Statistical analyses.

The results are expressed as means ± standard deviations (SD). Data were collected from triplicate experiments and are representative of at least three independent experiments. Groups were compared using one-way analysis of variance (ANOVA) in GraphPad Prism (San Diego, CA, USA). The level of significance was set at a *P* value of <0.05. Two-way ANOVA was used when appropriate. Mouse survival was compared using the Mantel-Cox version of the log-rank test.

### Data availability.

The transcriptome data files were uploaded into the Sequence Read Archive (SRA) of the National Center for Biotechnology Information (NCBI) (BioProject identifier [ID] PRJNA720971).
