# Supplementary material for: PGRMC1 Exerts Its Function of Anti-Influenza Virus in the Central Nervous System
Source: Microbiol Spectr. 2021 Sep 29;9(2):e00734-21. doi: 10.1128/Spectrum.00734-21 (PMC8557870; doi:10.1128/Spectrum.00734-21)
Supplement: SUPPLEMENTAL FILE 1 — Supplemental material. Download SPECTRUM00734-21_Supp_1_seq2.pdf, PDF file, 0.1 MB [file spectrum00734-21_supp_1_seq2.pdf]

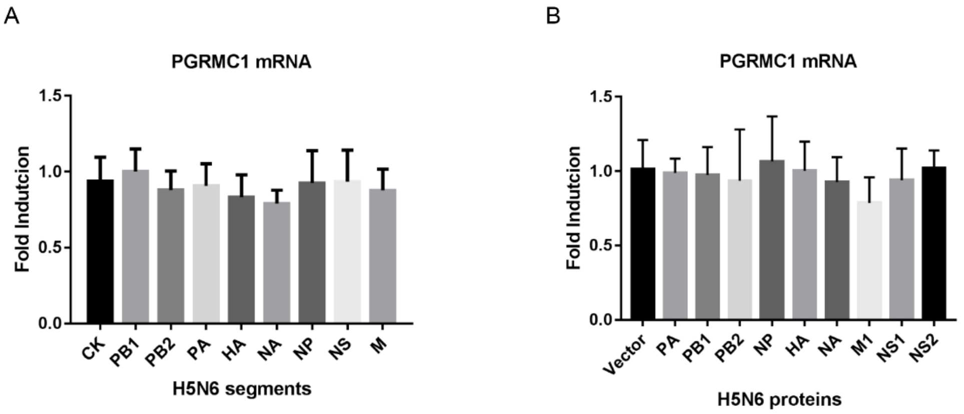

Figure S1. No single IAV JX gene segment or protein was able to significantly down-regulate PGRMC1. Eight viral gene segments were individually cloned in the pHW2000 expression vector and nine proteins (PA, PB1, PB2, NP, HA, NA, M1, NS1, NS2) were cloned in the p3×Flag-CMV-14 vector. U251 cells were transfected with the above plasmids and empty vectors. After 48 h, PGRMC1 mRNA were determined using qRT-PCR.

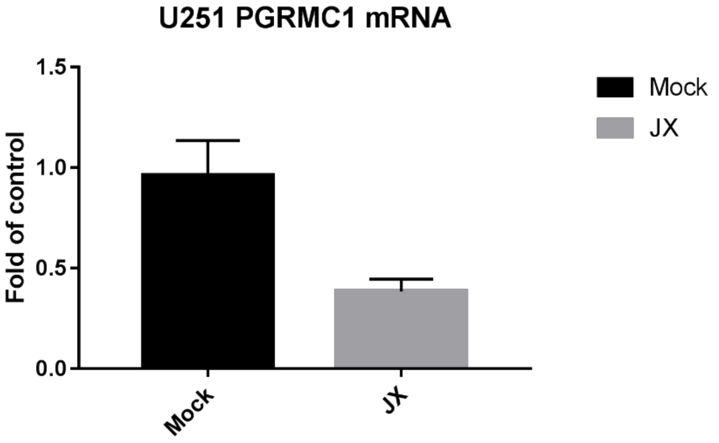

Figure S2. H5N6 down-regulated the expression of PGRMC1 in brain of mice. Mice (n = 3) aged 4-6 weeks were intranasally infected with 50 μL of 105 TCID50 H5N6 virus.

Mice in each group were euthanized at 5 dpi. PGRMC1 mRNA in lung was determined through qRT-PCR.
